# Personalizing health care: feasibility and future implications

**DOI:** 10.1186/1741-7015-11-179

**Published:** 2013-08-13

**Authors:** Brian Godman, Alexander E Finlayson, Parneet K Cheema, Eva Zebedin-Brandl, Inaki Gutiérrez-Ibarluzea, Jan Jones, Rickard E Malmström, Elina Asola, Christoph Baumgärtel, Marion Bennie, Iain Bishop, Anna Bucsics, Stephen Campbell, Eduardo Diogene, Alessandra Ferrario, Jurij Fürst, Kristina Garuoliene, Miguel Gomes, Katharine Harris, Alan Haycox, Harald Herholz, Krystyna Hviding, Saira Jan, Marija Kalaba, Christina Kvalheim, Ott Laius, Sven-Ake Lööv, Kamila Malinowska, Andrew Martin, Laura McCullagh, Fredrik Nilsson, Ken Paterson, Ulrich Schwabe, Gisbert Selke, Catherine Sermet, Steven Simoens, Dominik Tomek, Vera Vlahovic-Palcevski, Luka Voncina, Magdalena Wladysiuk, Menno van Woerkom, Durhane Wong-Rieger, Corrine Zara, Raghib Ali, Lars L Gustafsson

**Affiliations:** 1 Department of Laboratory Medicine, Division of Clinical Pharmacology, Karolinska Institutet, Karolinska University Hospital Huddinge, SE-141 86, Stockholm, Sweden; 2 Strathclyde Institute of Pharmacy and Biomedical Sciences, University of Strathclyde, Glasgow, UK; 3 National Institute for Science and Technology on Innovation on Neglected Diseases, Centre for Technological Development in Health, Oswaldo Cruz Foundation (Fiocruz), Rio de Janeiro, Brazil; 4 King’s Centre for Global Health, Global Health Offices, Weston Education Centre, Cutcombe Road, London SE5 9RJ, UK; 5 Sunnybrook Odette Cancer Centre, 2075 Bayview Avenue, Toronto, ON, Canada; 6 Hauptverband der Österreichischen Sozialversicherungsträger, 21 Kundmanngasse, AT-1031, Wien, Austria; 7 Institute of Pharmacology and Toxicology, Department for Biomedical Sciences, University of Vienna, Vienna, Austria; 8 Osteba Basque Office for HTA, Ministry of Health of the Basque Country, Donostia-San Sebastian 1, 01010, Vitoria-Gasteiz, Basque Country, Spain; 9 NHS Tayside, Kings Cross, Dundee DD3 8EA, UK; 10 Department of Medicine, Clinical Pharmacology Unit, Karolinska Institutet, Karolinska University Hospital Solna, SE-17176, Stockholm, Sweden; 11 Pharmaceutical Pricing Board, Ministry of Social Affairs and Health, PO Box 33, FI-00023 Government, Helsinki, Finland; 12 Austrian Medicines and Medical Devices Agency, Traisengasse 5, Wien, Austria; 13 Public Health & Intelligence Strategic Business Unit, NHS National Services Scotland, Edinburgh EH12 9EB, UK; 14 Centre for Primary Care, Institute of Population Health, University of Manchester, Manchester M13 9PL, UK; 15 NIHR Greater Manchester Primary Care Patient Safety Translational Research Centre, Manchester M13 9PL, UK; 16 Unitat de Coordinació i Estratègia del Medicament, Direcció Adjunta d'Afers Assistencials, Catalan Institute of Health, Barcelona, Spain; 17 London School of Economics and Political Science, LSE Health, Houghton Street, London WC2A 2AE, UK; 18 Health Insurance Institute, Miklosiceva 24, SI-1507, Ljubljana, Slovenia; 19 Medicines Reimbursement Department, National Health Insurance Fund, Europas a. 1, Vilnius, Lithuania; 20 INFARMED, Parque da Saúde de Lisboa, Avenida do Brasil 53, 1749-004, Lisbon, Portugal; 21 Liverpool Health Economics Centre, University of Liverpool, Chatham Street, Liverpool L69 7ZH, UK; 22 Kassenärztliche Vereinigung Hessen, 15 Georg Voigt Strasse, DE-60325, Frankfurt am Main, Germany; 23 Norwegian Medicines Agency, Sven Oftedals vei 8, 0950, Oslo, Norway; 24 Clinical Programs, Pharmacy Management, Horizon Blue Cross Blue Shield of New Jersey, Newark, USA; 25 Republic Institute for Health Insurance, Jovana Marinovica 2, 11000, Belgrade, Serbia; 26 State Agency of Medicines, Nooruse 1, 50411, Tartu, Estonia; 27 Department of Healthcare Development, Stockholm County Council, Stockholm, Sweden; 28 HTA Consulting, Starowiślna Street, 17/3, 31-038, Cracow, Poland; 29 Public Health School, The Medical Centre of Postgraduate Education, Kleczewska Street, 61/63, 01-813, Warsaw, Poland; 30 NHS Greater Manchester Commissioning Support Unit, Salford, Manchester, UK; 31 National Centre for Pharmacoeconomics, St James's Hospital, Dublin 8, Ireland; 32 Dental and Pharmaceuticals Benefits Agency (TLV), PO Box 22520 Flemingatan 7, SE-104, Stockholm, Sweden; 33 University of Glasgow, Glasgow, Scotland, UK; 34 University of Heidelberg, Institute of Pharmacology, D-69120, Heidelberg, Germany; 35 Wissenschaftliches Institut der AOK (WIDO), Rosenthaler Straße 31, 10178, Berlin, Germany; 36 IRDES, 10 rue Vauvenargues, 75018, Paris, France; 37 KU Leuven Department of Pharmaceutical and Pharmacological Sciences, 3000, Leuven, Belgium; 38 Faculty of Pharmacy, Comenius University and Faculty of Medicine, Slovak Medical University, Bratislava, Slovakia; 39 Unit for Clinical Pharmacology, University Hospital Rijeka, Krešimirova 42, 51000, Rijeka, Croatia; 40 Ministry of Health, Republic of Croatia, Ksaver 200a, Zagreb, Croatia; 41 Dutch Institute for Rational Use of Medicines, 3527 GV, Utrecht, Netherlands; 42 Institute for Optimizing Health Outcomes, 151 Bloor Street West, Suite 600, Toronto, ON M5S 1S4, Canada; 43 Barcelona Health Region, Catalan Health Service, Esteve Terrades 30, 08023, Barcelona, Spain; 44 INDOX Cancer Research Network, Cancer Epidemiology Unit, University of Oxford, Oxford, UK

**Keywords:** Biomarkers, Drug development, Genomics, Genotyping, Healthcare policy, Pharmacogenetics precision medicine, Personalized medicine, Health authorities, Rational use of medicines, Reimbursement, Targeted treatments

## Abstract

Considerable variety in how patients respond to treatments, driven by differences in their geno- and/ or phenotypes, calls for a more tailored approach. This is already happening, and will accelerate with developments in personalized medicine. However, its promise has not always translated into improvements in patient care due to the complexities involved. There are also concerns that advice for tests has been reversed, current tests can be costly, there is fragmentation of funding of care, and companies may seek high prices for new targeted drugs. There is a need to integrate current knowledge from a payer’s perspective to provide future guidance. Multiple findings including general considerations; influence of pharmacogenomics on response and toxicity of drug therapies; value of biomarker tests; limitations and costs of tests; and potentially high acquisition costs of new targeted therapies help to give guidance on potential ways forward for all stakeholder groups. Overall, personalized medicine has the potential to revolutionize care. However, current challenges and concerns need to be addressed to enhance its uptake and funding to benefit patients.

## Introduction

### General

Considerable variability exists in how individual patients respond to pharmacological treatments. Differences in patients’ individual make-up arising from genetic, biological, behavioral and environmental factors are seen as causes of this variability [1-15]. Patients’ genomes account for an estimated 20% to 95% of the variation in drug disposition [16,17]. This variability translates into differences in clinical outcomes including therapeutic benefit and side-effects [14,15,18-22]. As a result, different dosing regimens may be needed. However, current treatment regimens still tend to use ‘general or average’ doses [3-5,9,13,14,22-25], calling for a more tailored approach in the future [14,26].

Some physician groups already specify different treatments and doses taking into account factors such as patients’ ages, gender, family history and current co-morbidities [5,27], and this is expected to continue. For instance, tamoxifen for many years has been standard treatment for patients with breast cancer with estrogen receptor sensitivity [26,28-31], but not when these receptors are absent. Omalizumab, a recombinant humanized antibody to IgE, is only recommended for patients with asthma uncontrolled with chronic steroids and who have convincing IgE-mediated asthma [32], with serum IgE levels used to determine subsequent doses. However, there continues to be controversy surrounding its effectiveness in clinical practice and its cost-effectiveness [33].

The recognition of the complexity of the various biological systems involved in different diseases [14,34] helps explain why there are a high number of non-responders to certain drugs (as high as 30% to 70%, or more) [1,5,35-37]. This in turn translates into an increase in the number of patients needed to treat, leading to physicians adopting trial-error paradigms when treating patients [27]. Inter-individual variability in patients’ responses can also increase adverse events and reduce effectiveness, or both [1,4,5,17,18,22,26,38-43], leading to sub-optimal care and adding to the costs of care. Increasing knowledge of the complexity of biological systems is also challenging drug development policies. This helps to explain why, between 2007 and 2010, for instance, 90% of drugs failing during phase II tests or in submission to the US Food and Drug Administration (FDA), failed due to either a lack of efficacy (66%) or a link to safety concerns (21%) [16,22,44-48].

As the knowledge of biological systems grows, drug pipelines should become more productive as well as improve subsequent patient care [8,9,13,35,36,49,50]. Between 1976 and 2005, 28 drugs were withdrawn from the market in the US due to idiosyncratic serious side-effects including hepatotoxicity, nephrotoxicity and rhabdomyolysis [18,51,52]. Specific examples include cerivastatin and mibefradil, both of which had favorable benefit-risk profiles at market authorization, but their use in clinical practice, coupled with physicians ignoring recommended guidance, caused their withdrawal [1,53]. Perhexiline was highly effective in treating angina; however it was associated with severe and unacceptable hepatotoxicity, leading to its withdrawal. This did not happen in Australia and New Zealand, where usage was linked to pre-treatment phenotyping and therapeutic drug monitoring. This is because side-effects, including neuropathy, had been linked to patients being poor or intermediate metabolizers of *CYP2D6*[5]. As a result, instigating routine pharmacogenetic testing at its launch may have prevented its withdrawal [5,54-57]. Dosing of thiopurines such as azathioprine according to patients’ thiopurine methyl transferase status can reduce subsequent drug-induced morbidity among patients with rheumatologic and inflammatory bowel disorders [19-21,58], potentially reducing discontinuation [20,21,58]. In addition, measuring thiopurine methyl transferase levels to substantially dose thiopurines can reduce the time taken to adequate dosing, helping with subsequent remission [59].

A different example is natalizumab, which was approved in 2004. In patients with relapsing remitting multiple sclerosis, natalizumab significantly reduced the number of relapses and the development of new inflammatory lesions [60,61]. However, natalizumab was withdrawn soon after its launch due to the development of progressive multifocal leukoencephalopathy (PML) resulting from reactivation of JC virus [60,61]. This was a major concern as PML is a devastating condition, leaving survivors with serious impairment. Natalizumab became available again in Europe in 2006 under strict prescribing regulations [60,61]. Programs are ongoing to investigate whether seropositivity for JC virus antibodies will help accurately predict the development of PML [60] as well as improve understanding of the risks of patients developing PML if they remain seronegative to JC virus and, alternatively, the risks of developing PML if they convert from seronegativity to seropositivity. As a result, these tests have the potential to improve the benefit:risk ratio. This continual re-evaluation is important to avoid unpredictable events.

These examples illustrate the opportunities and challenges that are concomitant with greater knowledge about disease progression and treatments.

### Resource issues

Adverse drug reactions (ADRs) add to the costs of healthcare by increasing hospital admissions [62-70]. Average treatment costs for a single ADR in Germany have been estimated at approximately €2,250, equating to €434 million per year [63]. The cost of emergency-related admissions in the UK because of ADRs has been estimated at GBP£2 billion annually [64]. It is estimated that more than 2 million people are hospitalized annually in the US through serious adverse events [22,71], and hospital admissions related to warfarin complications costing on average US$10,819 per patient [72]. Overall, the cost of drug-related morbidity and mortality exceeded US$177 billion in the US in 2000 [35]. Hospital admissions accounting for nearly 70% of the total costs followed by long-term care admissions (18%) [22,73]. ADRs are also a challenge to healthcare institutions in low income countries [68].

Improved knowledge of pharmacogenomics could potentially reduce ADRs [15,18] through, for instance, improved identification of host genetic factors predisposing patients to increased toxicity to certain drugs [15,16,18,22,38,39,74,75].

In Europe, the current financial crisis makes resource issues especially important. Pharmaceutical expenditure has been growing at a faster rate than other components of ambulatory care [76-83], equating to 50% in real terms among Organisation for Economic Co-operation and Development countries between 2000 and 2009 [83,84]. As a result, pharmaceutical expenditure has become the largest or equaling the largest cost component in ambulatory care in many countries [78-83,85]. New premium-priced drugs, especially new biological drugs at US$100,000 to US$300,000 per patient per year or more, are adding to these pressures, challenging the ability of society to continue to provide equitable and comprehensive healthcare [82,83,86-88]. Some countries are already not reimbursing new premium-priced drugs [89,90], which is not in the best interest of any key stakeholder group.

Consequently, it is attractive to tailor treatments and resources to patients according to their genetic, medical and behavioral factors to achieve the greatest health gain, minimizing waste and maximizing the number of patients needed to harm [4,5,8,9,14,16,18,22],[23,36,45,74,91,92]. This should result in healthcare systems maximizing the improvement in the health of their patients with available resources. It may also lead to the stratification of treatments according to their health gain becoming a major factor in future reimbursement considerations for new premium-priced drugs.

We are already seeing capacity building in genomics medicine and molecular diagnostics growing across countries, including Sri Lanka and Asia-Pacific countries [93-95]. This is likely to continue with new developments and resources.

### Objectives and definitions

Personalized medicine and personalized healthcare are not new concepts [27,44,47,96,97]. Personalized medicine was initially established in oncology, where new therapeutic concepts could be developed upon the precise description of disease-specific mutations. As a result, new strategies evolved such as targeted therapies and signal interception-based therapies. However, for the purposes of this paper, personalized medicine not only refers to the choice of therapeutic strategies in terms of direct target selection but also involves a degree of pharmacogenomics and genetic testing to improve patient care [16], agreeing with other authors [26,98,99].

We recognize that the development of personalized approaches is complex. This is illustrated by recent research in breast cancer, suggesting that this cancer consists of many different types of tumors [100]. Greater knowledge of this diversity should lead to developments that improve the sensitivity and specificity of prognostic and diagnostic biomarkers; this should also lead to more effective treatments [11,100-102]. We believe greater targeting of treatments has the potential to revolutionize healthcare delivery through improved effectiveness of treatments and reduced side-effects and associated costs, as well as reducing the number of product withdrawals [18,26,27]. Current knowledge is already resulting in the growth of medicines that require genetic testing before administration [4,5,11,13,15,19,21,45],[46,49,86,91,103-106]. This will continue given the number of targeted treatments as well as genomic tests in development [45,107]. We also expect to see more accreditation of laboratories performing genomic testing of specific molecular genetic traits to improve the interpretation of laboratory results [16]. This accreditation will address concerns where there has been variation in test results depending on the detection methodologies used [13,108]. The sensitivity and specificity of pharmacogenetic tests is important as this will affect their cost-effectiveness and subsequent utilization [98,99].

However, as in many growing fields, the promises associated with pharmacogenomics have not always translated into appreciable improvements in patient care [14,22,47]. This includes the clinical utility of genomic tests for which a national expert panel (Evaluation of Genomic Applications in Practice and Prevention; EGAPP) in the US only recommended one of the four tests initially evaluated for routine use, with the need for more evidence in two [107,109,110]. It is recognized that there are a number of barriers that need to be addressed before pharmacogenomics will be part of routine clinical care [27]. These include a redirection of drug development towards tailored therapies [23,36,92], which is already happening [45,86,111]. Major issues regarding the funding of genetic and biomarker testing, especially high cost tests, also need to be addressed given the current and diverse funding structures between ambulatory and hospital care, and the fact that traditional diagnostic tests have typically been low priced [35,45,74,75]. Instigation of projects such as EGAPP will help improve the systematic way in which tests are evaluated for their potential clinical utility to help with funding decisions. Other barriers include addressing health authority and health insurance (‘payer’) concerns that companies will seek high prices for new targeted treatments through seeking orphan status [82,86-88,112-116]. There may also need to be changes during drug development processes, including clinical trials, where sub-populations will continue to shrink to fulfill licensing and reimbursement requirements. This includes better identification of patients likely to have an improved benefit:risk profile because of their pharmacogenetic profile.

Consequently, the objective of this review paper is to integrate current knowledge about the value of biomarkers and prognostic tests to improve patient care, as well as potential concerns, from a payer’s perspective. This is because published articles have generally not been written with this in mind. As a result, we hope this paper will provide guidance to all key stakeholder groups on potential ways to enhance future utilization and funding of new personalized approaches. This will be achieved by reviewing the current situation regarding personalized medicine, principally based on peer-reviewed papers, building on comments in the background; appraising key funding, organizational and healthcare issues that need to be addressed; and suggesting potential ways forward for all key stakeholder groups to enhance funding and utilization of new diagnostic and prognostic tests as well as new targeted drug treatments through an iterative process involving the co-authors.

We will consider the need to distinguish between genetic tests that demonstrate a particular patient has susceptibility to a given disease and developments that help determine a patient’s responsiveness to a given drug and/or the potential for adverse reactions [5,10,117].

We are aware of the considerable controversies surrounding unregulated direct-to-consumer (DTC) genetic testing [118] and we will briefly mention this.

## Review

The findings are consolidated under various headings, including general considerations, pharmacogenomics, biomarkers, challenges and concerns, and key issues for healthcare funding bodies to address.

### General considerations

Multiple definitions have been assigned to personalized medicine, including stratified medicine. Essentially all definitions include targeting of diagnostic or treatment approaches to improve the future care of patients [2,8,16,23,26,27,45,50],[98,119,120].

As we explore the molecular pathophysiology of different diseases, we find increasing examples of genetic differences as explanations for inter-individual variability in drug responses [1-5,8-11,16,22,37,47]. For example, the efficacy and safety of codeine is influenced by *CYP2D6* polymorphisms, explaining why slow *CYP2D6* metabolizers lack an analgesic effect with codeine and why ultra-rapid metabolizers may experience adverse effects on therapeutic doses [16,22,38,39]. Greater knowledge allows the possibility to redefine patient subgroups for drugs to enhance their effectiveness and/or reduce their toxicity. Consequently, improved knowledge of biomarkers will enrich the management of diseases from prevention to treatment depending on the availability of targeted therapies. Greater knowledge should also enhance the use of existing therapies, reducing reliance on new premium-priced therapies. Whether this happens remains to be seen, especially given that, despite many decades of scientific advances, only a few genotyping or phenotyping tests are currently being used routinely in clinical practice [4,5,10,11,13,16,21,36],[105,121,122]. This is because of increasing knowledge and awareness that a given patient’s genomic and phenotypic make-up is appreciably more complex than initially believed, as well as the influence of environmental factors [1,9-12,16,36,49,117,122]. As a result, different approaches are required to separate diseases into different subgroups. These include new technology platforms and mathematical models of different approaches and consequences, including system biology approaches that replicate diseases, to truly realize ‘personalized medicine’ [8,34,36,44,123]. This will be helped by the continued development of patient-centric, longitudinal and cross-institutional electronic health records containing genetic information and genomic test results, while ensuring patients’ privacy through appropriate data encryption and privacy protection [44,124].

### Pharmacogenomics and response and toxicity to drugs

Pharmacogenomics includes the identification of host genetic factors that influence drug absorption, metabolism and action at the receptor level, which could subsequently reduce patient numbers needed to treat and minimize toxicity [125,126]. There are a number of examples of pharmacogenomics being applied across diseases areas. These can be summarized into tests that are associated with increased response rates and those that predict toxicities to improve future care (Table 1).

**Table 1:** Examples of pharmacogenomics tests regarding responses or toxicities to drug treatments

**Disease area**	**Host genotype**	**Treatment**	**References**
***Optimizing drug efficacy***			
Chronic hepatitis C	*IL28B*	Pegylated interferon alpha, Ribavirin	[127-129]
Breast cancer, other tumors		Anthracyclines, poly(adenosine diphosphate-ribose) polymerase inhibition	[117,130-132]
***Preventing drug toxicities***			
HIV type 1	(HLA)-B*5701	Abacavir	[1,4,35,106,133]
Rheumatic and inflammatory bowel disorders	*Thiopurine methyl transferase* genotypes	Azathioprine	[19-21,58,59,133-135]
	HLA-B*58:01	Allopurinol	
Gastrointestinal cancers	Dehydropyrimidine dehydrogenase deficiency	5-fluorouracil	[136]
	*UGT1A1* polymorphism	Irinotecan	[137]

For example, independent genome-wide studies involving patients with chronic hepatitis C infection, who were treated with pegylated interferon alpha and ribavirin, showed an association between a variant in the host genotype of *IL28B* and drug response [127-129]. In view of this, genotyping for *IL28B* is increasingly undertaken in hepatitis C clinics [4]. Poly(adenosine diphosphate–ribose) polymerase (PARP) inhibitors are also showing promise in a subgroup of patients with triple negative breast cancer who have inherent defects in DNA repair. This makes this particular breast cancer a rational target for therapy based on PARP inhibition [138]. Models have also been developed, including four polymorphisms in the *AMPD1*, *ATIC*, *ITPA* and *MTHFD1* genes, to help predict response to methotrexate, leading to greater tailoring of treatment [139].

Pharmacogenomics has also been effective in helping to predict toxicities to treatment. Examples include (Table 1) testing prior to initiating abacavir in patients with HIV type 1. It is estimated that between 48% and 61% of patients with the human leukocyte antigen-B*5701 allele will develop a hypersensitivity reaction to abacavir, which can be life threatening if repeated, compared with to 0% to 4% of patients who do not have this allele [1,4,35,140]. This resulted in the FDA modifying the abacavir label to include a recommendation that patients should undergo allele testing before initiation [125]. There is also awareness that dihydropyrimidine dehydrogenase deficiency may need to be tested for in patients prior to initiation of 5-fluorouracil (infusion or oral tablets) for the management of their gastrointestinal cancer. Full dihydropyrimidine dehydrogenase deficiency can be fatal but rare in practice; however, partial deficiency is present in 3% to 5% of patients [136].

### Biomarkers to target treatment approaches

The National Cancer Institute in the US defines a biomarker as a biological molecule found in blood, other body fluids, or tissues that is a sign of a normal or abnormal process, or of a condition or disease [117,141]. Biomarkers are increasingly being used in the field of cancer as well as other disease areas (Table 2). They are also being investigated in the field of psychiatry including directing treatment approaches in patients with schizophrenia [142].

**Table 2:** Examples of tumor-specific biomarkers to determine eligibility for targeted therapy

**Disease**	**Biomarker**	**Drug**	**References**
Colorectal cancer	*KRAS*	Cetuximab, panitumumab	[1,2,11,13,27,86,98,108],[125,143,144]
Breast cancer	Estrogen receptor	Tamoxifen	[28-31]
Breast cancer	Human Epidermal Growth Factor Receptor 2 (HER 2)	Trastuzumab, pertuzumab, lapatinib, trastuzumab emtansine (TDM1)	[1,2,11,27,125,145-154]
Melanoma	*BRAF*	Vemurafenib, dabrafenib	[34,112,155-157]
Non-small cell lung cancer	Epidermal Growth Factor Receptor (EGFR)	Gefitinib, erlotinib, afatinib	[1,23,158-165]
Non-small cell lung cancer	Anaplastic Lymphoma Kinase (ALK) inhibitors	Crizotinib	[91,163,166,167]
Chronic myeloid leukemia	Philadelphia chromosome levels	Imatinib, nilotinib, dasatinib	[168,169]
CCR5-tropic HIV	CXCR4, CCR5 receptors	Maraviroc	[170]
Cystic fibrosis	*G551D* mutation	Ivacaftor	[88]

The importance of incorporating biomarkers into drug development is illustrated by gefitinib. Gefitinib was given conditional approval in 2003 for the treatment of chemorefractory metastatic non-small cell lung cancer. However, a large trial assessing the efficacy of gefitinib in an unselected patient population failed to show a survival benefit [158]. This led the FDA to re-label gefitinib restricting its use to patients that were already using the drug and benefiting from it. Subsequently, it became apparent that patients with tumors that have Epidermal Growth Factor Recepetor (EGFR) -activating mutations, which are present in 10% to 26% of non-small cell lung cancers, significantly benefited from gefitinib compared to standard chemotherapy [1,23,159-162]. As a result, the FDA altered the label of gefitinib to include its use in patients with tumors that are EGFR-activating mutation positive. These patients will also have increased responsiveness to the tyrosine kinase inhibitors erlotinib and afatinib (Table 2) [163-165]. Not surprisingly, with new ways to identify subgroups of tumors with response to specific drugs, it is becoming a prerequisite in oncology for companies to design clinical trials with genetic biomarkers [36,49,91,125,171,172].

Examples of targeted treatments in non-cancer areas include maraviroc for patients with HIV (Table 2). Maraviroc is only effective against CCR5-tropic HIV, and patients with viruses that use both the CXCR4 and CCR5 receptors for entry into the cell (dual/mixed tropic) will not respond [170]. Consequently, it should not be initiated in these patients. Awareness of the fact that the funding and prescribing of maraviroc may be challenged by the cost of testing, at up to US$1,960 per patient, resulted in the company covering these costs themselves [35]. However, the cost of this test was fully covered by insurance companies in the US within twelve months of its launch [35].

There has also been a search to identify easy-to-use biomarkers. Recently, a simple blood test to detect circulating tumor cells in patients with breast cancer has been reported to offer prognostic information [132]. Blood tests are also being developed to rapidly predict which patients will respond to anthracyclines or PARP inhibition [117,130-132]. Patient management is clearly being improved with the use of biomarkers with existing drugs. This, along with increasing knowledge of gene expression and aberrant signaling pathways [13,49,117,142,173-175], should increase the number of drugs that can be more rationally prescribed and dosed using biomarkers, and also broaden the use of established drugs.

### Challenges and concerns for routine use of diagnostic tests

There have been controversies and concerns regarding the routine use and funding of some pharmacogenetic tests. This is in view of their sensitivity, specificity, associated costs or a combination of these factors.

The EGAPP group [110] had concerns with three out of the first four tests evaluated [107,109]. These included tumor gene expression profiles to improve outcomes in defined populations of women with breast cancer, *CYP450* testing of drug metabolic capacity before treating adults with depression with selective serotonin re-uptake inhibitors and population screening for hereditary hemochromatosis [107,109,176]. There are ongoing debates regarding the utility of *CYP2D6* genotyping testing prior to initiating tamoxifen [5,28-31,177]. In November 2010, this resulted in the UK Medicines and Healthcare Products Regulatory Agency no longer recommending genetic testing prior to treatment with tamoxifen [5,178].

There are also continuing controversies surrounding genetic testing prior to initiation with either clopidogrel or warfarin. Studies suggest there is an increased risk of adverse cardiovascular outcomes if effective and safe drug concentrations of these drugs are not reached [4,5,125,179]. This includes in patients with allelic variants of the genes *CYP2C19* and *VKORC1*. This resulted in the FDA revising the label for clopidogrel in June 2009 to include a section on pharmacogenetics explaining that several CYP enzymes convert clopidogrel to its active metabolite and that the patient’s genotype for one of these enzymes (*CYP2C19*) could affect its activity [5]. More recently, a number of studies have reached different conclusions making the situation unclear [125,179-184]. As a result, the American College of Cardiologists in 2010 advised that the predictive value of pharmacogenetic testing prior to clopidogrel therapy is limited and the evidence base is insufficient to recommend routine testing [185]. This has been endorsed by two recent meta analyses, both of which failed to show a substantial or consistent influence of *CYP2C19* polymorphisms on subsequent cardiovascular events [184,186]. The Medicare Evidence Development & Coverage Advisory Committee, Centers for Medicare and Medicaid Services, had concerns with genotype-guided dosing of warfarin [109]. They suggested a genotype-guided test could still be used but should be accompanied by evidence development in view of the uncertainties involved [41,109,187]. As a result, they did not endorse routine pharmacogenetic testing prior to initiating warfarin. This may change with recent data suggesting that up to 50% of the variation in the dose of warfarin needed may be explained by genetic factors [188]. Recent studies also suggest that genetic information on *CYP2C9* and *VKORC1* is important both for the initial dose finding stage with warfarin as well as during maintenance therapy [188].

There is also continuing debate about the funding of *BRCA* testing and Oncotype DX in terms of their associated costs and cost-effectiveness in practice. This is illustrated by variable funding among private and public insurance plans in the US for *BRCA* testing for patients at high risk of developing breast cancer, exacerbated by charges of US$3,340 per patient for full sequence testing [121,189]. Nevertheless, the National Institute for Health and Care Excellence (UK) currently recommends that *BRCA1*/*2* testing should be offered to patients if the likelihood of detecting a mutation is greater than 20%, although many testing centers offer the test if the likelihood is between 10% and 20% [190,191]. Wider usage is currently difficult to endorse in view of the costs involved [191]. This may change with recent research showing that women in the UK diagnosed with triple-negative breast cancer under 50 should routinely be offered *BRCA1*/*2* testing to optimize subsequent treatment options that the genetic information makes possible [190,192]. However, such a move would result in an extra 1,200 tests a year in the UK [191]; although some of costs could be offset by reduced waste.

The Oncotype DX breast cancer assay is a 21-gene assay conducted on a patient’s tumor specimen to estimate the risk of recurrence post surgery specifically in patients with hormone-positive, lymph node-negative breast cancer. It can also provide further information on the benefits patients will derive from postoperative chemotherapy. The impact of the Oncotype DX score was evaluated prospectively and led to treatment changes in 30% of cases [193]. Both the American Society of Clinical Oncology and the US National Comprehensive Cancer Network guidelines endorse its use in early stage hormone-positive breast cancer [121,194]. Despite these recommendations, and two Canadian studies showing that molecular classification with this test is clinically useful and cost-effective [195,196], funding for this assay remains variable among the Canadian provinces. This can be largely attributed to the average cost of a test at CA$4,000 [194]. In 2010, British Columbia began a registration study for the 21-gene assay. However, it was restricted to node-negative cases and, until recently, was active only in the main Vancouver clinic. In 2010, Ontario started funding the 21-gene assay more consistently, with prior authorization needed for out-of-country cases. Recently, Quebec’s public system has also started funding an increasing number of these tests [194].

There is also an Oncotype DX colon cancer assay, which is a 12-gene assay to predict recurrence after resection of stage II and III colon cancer. This assay has been validated in a number of studies [197-199]. When conducted on tumor specimens of appropriately selected patients with stage II colon cancer, there was a 17% reduction in the use of postoperative chemotherapy and, similar to breast cancer, the Oncotype Dx colon cancer assay led to a change in a patient’s treatment in a third of cases [200]. The test is currently not funded by any of the Canadian provinces, but has been funded through Centers for Medicare and Medicaid Services in the US since September 2011.

The costs of pharmacogenomics testing are envisaged to fall appreciably with their increasing use [44,201]. This should enhance their funding and use provided there is robust evidence including details of their sensitivity and specificity. Reimbursement will also be enhanced if the current heterogeneity in funding systems can be resolved where pertinent [27].

### Future research priorities including strategies between the US Food and Drugs Administration and the European Medicines Agency

These case histories illustrate why it is crucial for researchers and commercial organizations to obtain data from trials demonstrating any association between biomarkers and disease outcomes to enhance future endorsement and funding of diagnostic tests. The same holds for new targeted treatments. Alongside this, a cohesive vision of what personalized medicine will constitute must be developed. The instigation of groups such as the Personalized Medicine Consortium and European Medical Research Council and the combining of research group activities should help with this [1,16,46,47]. European initiatives such as Information and Telecommunication for the Future of Medicine [202] should also help with study design through developing methodologies incorporating multiple forms of evidence such as those from different genetic databases [27]. This includes the findings from the 1000 Genomics Project Consortium [203]. Implementation of guidance at the public health level such as the Public Health Genomics European Network II guidelines for the provision, quality assurance and use of pharmacogenomic tests [27] should help enhance the adoption of new tests in a rational manner.

Pharmacogenomic research should be strengthened by bodies such as the International HapMap Consortium, the 1000 Genomes project [16], the Mutanom project (German National Genome Research Network - NFGN - combined with Integrated Genomic Research Network - IG) [44], Pharmacogenomics Research Network [46] and the International Cancer Genome Consortium [47].

It is recognized that full targeting of diagnostic and treatment approaches will require appreciable understanding of the genetic background for different diseases and patient populations, rather than just the expression patterns of single gene associations. This has been demonstrated by the variable predictive yield of genome-wide association studies to date [14,18,46,47,171]. As mentioned, this can be explained by the complex nature of biological systems, which have shown to operate in far more complex ways than originally thought.

Future developments may include improved translation of single and combined biomarker test information. They may also include developments in technology platforms, mathematical models and systems biology [2,17,36,46], thereby reducing the heterogeneity of currently treated populations through smaller subgroups [13,37,77]. However, this will require more extensive basic and clinical research than is currently being funded [9,204].

It is also recognized that clinical trials to evaluate new tests and/or targeted treatments may be complex and costly, and may also pose serious organizational and ethical problems if there are multiple subgroups with different treatment strategies [98,99]. This calls for new study designs, potentially including sequential testing. One way forward could be to have clinical trial and observational study evidence combined with systems biology modeling such that multiple trials validate the mathematical models produced. These can subsequently be used to predict treatment effect for individual patients and their tumors [34,44]. However, such studies need specific objectives including the prospective definition of diagnostic, screening or prognostic biomarkers alone and/or in combination before any studies are undertaken [28,98,99,117,162,205]. Innovative funding strategies may also be needed to accelerate the introduction of new valued targeted treatment approaches until their costs come down [44,201]. The combination of prospective clinical trials and observational studies may also accelerate the translation of clinical research results into routine medical practice [2].

It will be increasingly important for the European Medicines Agency and the FDA to collaborate on the development and establishment of harmonized guidelines for genotyping and biomarker testing, and their incorporation into future targeted treatments, to guide companies [133]. This could include standardizing trial data documentation. The importance of this is emphasized by up to 50% of current clinical pipelines among leading companies include targeted or stratified medicines [45]. In addition, as mentioned, in oncology it is becoming a prerequisite for pharmaceutical companies to design clinical trials wich include biomarkers.

### Key issues for healthcare and funding bodies

#### General

As this field evolves, the clinical utility in shaping patients’ treatment should become realistic [2]. However, several issues need to be addressed.

Key issues include clearer co-ordination between the various bodies responsible for funding of care and those evaluating new treatment approaches [13]. Improved co-ordination should help to assimilate more rapidly proven developments of value into routine clinical practice. This is happening, for example in France, with the simultaneous assessments of new diagnostic technologies by the Commission Nationale d’Evaluation des Dispositifs Médicaux et des Technologies de Santé, and the evaluation of new drugs and their associated diagnostic test by this institution together with the Transparency Commission [35].

There also needs to be effective strategies that address current concerns among health authority and health insurance personnel regarding personalized medicine. This is because there are currently few examples of pharmacogenetic tests being integrated into routine care despite the initial optimism. In addition, the advice on whether to fund specific tests has changed on a number of occasions as more research data becomes available. Accommodating these costs will not be easy, especially with growing resource pressures [1,35]. However, there is willingness among payers to consider new tests and treatments given the resources that are currently being wasted as clinicians try different treatment approaches as well as the costs of treating ADRs [27,62-73]. The costs for any pharmacogenomic tests associated with new targeted treatments need to be made explicit in any health technology assessment of new drugs, including the potential budget impact.

As a result, there are a number of medical, ethical, legal, social, economic and organizational issues that need to be considered as the field of personalized medicine grows (modified from [27,109]):

*Medical issues*

• Improvements in clinical effectiveness through tailoring treatments including their impact on length and quality of life as well as number of patients needed to treat

• Improvements in drug safety profiles/reductions in adverse drug reactions increasing the number of patients needed to harm rates

• Relevance of surrogate results (diagnostic technologies)

• Opportunities for preventive measures and interventions

• Proportion of patents affected/re-classified

• Need for post-marketing follow-up (post-introduction assessment) and not just pharmacovigilance

• Improved knowledge of pharmacogenomics among physicians

*Legal issues*

• Reassessing existing drugs and other technologies

• Redefining existing regulatory policies

• Need for including biomarkers that support indications and clinical decision making

• Maintenance of citizens’ autonomy

• Legal liability associated with targeted tests

• Protection of any patient information generated

• Whether professional ethical guidelines become statutes or mandatory guidelines

• Harmonization of laws in different contexts

• Patients’ autonomy

• Advertising - particularly direct-to-consumer advertising

• Harmonization of free movement of services to avoid or reduce citizens’ misconceptions and potentially unlawful practices

*Economic issues*

• Reduction in costs to healthcare systems with greater personalized approaches

• Who pays for diagnosis - healthcare systems, manufacturers or patients? This especially with current fragmentation of care and budgets

• Budget impact of new technologies and other considerations for reimbursement and funding including cost and Quality Adjusted Life Year considerations

• Whether the inclusion of biomarkers will lead to more clearly defined subpopulations and indications for reimbursement (in addition to regulatory considerations)

• Potential changes in reimbursement considerations and policies with smaller populations and targeted treatments

• Redefining the conditions for orphan status for new targeted treatments

• Financial incentives for citizens; active role of the citizen in his/her own health and wealth

• Co-development of drugs and genome-based diagnostics that more tightly define indications or subpopulations - requirements for approval and/or incentives for reimbursement at premium prices

*Ethical issues*

• Change in the concept of health and disease (prediction)

• Ownership of the information (not only genomic)

• Sufficient understanding to justify population-based genome sequencing

• Accessibility of diagnostic tests and targeted treatments within and across countries with companies seeking ‘orphan status’ for new targeted treatments

• Patients’ understanding and patients’ role in future decision making

• Human dignity - potential for stigmatization and discrimination

• Human integrity - how this affects moral convictions, preferences and commitments

*Social issues*

• Empowerment and increasing autonomy of patients and their relatives

• Stigmatization of certain subpopulations according to their genomic, clinical and environmental data

• Current technology makes internationalization of data possible

• Policies to promote the implementation of personalized health services:

○ people and subgroups involved

○ support required

○ costs involved

○ people’s reaction for or against such services (qualitative and quantitative research)

• Legal barriers concerning different reimbursement and pricing policies that have an impact on the implementation of personalized healthcare services and products. This can cause inequity or unequal access to new technologies if not addressed

*Organizational issues*

• Emphasis on wellness and disease prevention

• Change in health services paradigm with primary care and public health playing a greater role with greater stratification of patients resulting in potential changes in work- and patient-flow processes

• Greater patient empowerment and shared decision-making:

○ Scheduled time dedicated to patients

○ Healthcare professional training on genomics including health literacy among providers

○ Management of genomic information and its consequences

○ Acquisition of shared decision-making skills

• Potential centralization of diagnostic services

• Potential decentralization of decision-making processes

• Monitoring of physician adherence to any tightly defined subpopulations; potential ways to enhance adherence where concerns

• Funds made available for data protection and complex computing systems

Overall, the medical, ethical, legal, social and economic challenges for personalized medicine are not unlike the scientific uncertainty, assessment, cost-effectiveness and access issues affecting traditional medicines. However, diverse ethical and social principles and their interpretation often lead to disparate views on the safety, equity and desirability of personalized therapy. For example, publicly directed genetic testing including newborn screening is being challenged to demonstrate, on the one hand, that informed consent, confidentiality and information accuracy are adequate and, on the other hand, that public and private good do result from identifying genetic abnormalities [206].

In addition, there must be discussion whether identification of a genetic predisposition, regardless of manifestation, could lead to denial of healthcare, insurance, employment or educational opportunities [207]. In terms of psychiatric conditions, there must also be a debate on how Health Technology Assessment (HTA) can balance the harm of stigmatization and discrimination based on a genetic sequence potentially related to a mental illness with the availability, affordability and effectiveness of prevention or treatment [208].

A key ethical concern going forward is equitable access to personalized therapies, which may affect drug development decisions. There may be, for example, fewer incentives for companies to develop drugs for ‘less profitable’ genotype groups [209]. If such practices increasingly become the norm, authorities will need to develop policies that redress this balance [119]. Finally, while HTA continues to adapt and evolve in its assessment and evaluations to personalized medicines, patients may experience unequal access where public and private drug coverage differs or in developing economies where priority may be given to population-based therapies. These issues will need addressing.

There are a number of potential ways forward for all the six main stakeholder groups to enhance the utilization and funding for new diagnostic or prognostic tests and treatment approaches that address the key issues highlighted above. These can be broken down into general issues as well as key issues pre-, peri- and post-launch [116,210].

*Potential issues for key stakeholder groups*

The first stakeholder group consists of governments, health authorities and health insurance agencies [3,5,27,35,83,97-99,107,109],[116,164,191,205,210-227]. The major issues for this group to consider include:

*General*

• To instigate Pan-European central, online, open-access repositories of biomarker and potential genomic tests of personalized therapy including their clinical utility and therapeutic implications. The data should be made readily and openly available to all key stakeholder groups.

• To establish respected groups in each country that can assess the value of new genetic tests prior to and during reimbursement or funding discussions. This builds on current activities in France, the National Institute for Health and Care Excellence, UK, the UK Genetic Testing Network and the Medicines and Healthcare Products Regulatory Agency in the UK, and the EGAPP working group in the US. This also builds on developments among HTA bodies (below).

• To introduce stricter definitions of orphan drug status to reduce the number of targeted drugs seeking this definition and their anticipated high acquisition costs, that is, 5 out of 100,000 rather than the current 5 out of 10,000 (below).

• To explore collaborative opportunities with groups such as the European Union Personalised RNA Interference to Enhance the Delivery of Individualised Cytotoxic and Targeted therapeutics consortium, and other European bodies, to deliver education to providers, practitioners and patients. This would address some of the complexities and misunderstanding that exists among key stakeholder groups regarding personalized medicine.

• To establish and support networks of professional medical institutions including Drug and Therapeutic Committees to promote critical drug evaluation peri-launch and scientifically founded recommendations. This also includes groups to assess the sensitivity and specificity of new diagnostic and prognostic tests and the implications across populations building on, for instance, the classification criteria developed by the EGAPP working group.

• To evaluate new ways of organizing care with personalized medicine placing particular emphasis on wellness and disease prevention replacing hospital-centerd care provision. This includes increased time between patients and physicians in primary care to fully explain the findings from any test to sufficiently empower patients in their decision making.

• To fully consider the legal consequences of personalized care including citizens’ autonomy, legal liability and the protection of any information generated.

• To refine new models of care broken down by pre-, per- and post-launch activities that enhance the utilization of new diagnostic technologies and new targeted treatments that can improve the care of patients.

Pre-launch

• To extend current Horizon Scanning, early assessment and alert systems as well as budget impact analyses to include new diagnostic and prognostic biomarkers and genetic tests. The objective is to ensure that independent information regarding the clinical utility of new tests, including issues surrounding their sensitivity and specificity as well as their overall predictive value, including data on the extent of false positives and false negatives, is available when new diagnostic approaches and new drugs are being considered for reimbursement. This may mean working initially with limited evidence while new data is generated. Such services can build on the activities of International Networks and EuroScan as well as Horizon Scanning activities in for instance Germany, Italy, Sweden and the UK. This should include an assessment of the likely budget impact of new diagnostic and prognostic approaches as well as new targeted treatments, including any costs avoided. It should also be ascertained beforehand whether tissue samples can be analyzed locally, for example, tissue samples have to be sent from Scotland to the US before initiating treatment with maraviroc, adding to the cost of treatment.

• As part of this, to initiate early dialogue with groups such as the European Network for HTA, country HTA bodies and the European Medicines Agency, as well as groups developing mathematical models and system biology approaches to interpret the findings from pharmacogenomics studies and their implications for subsequent patient care.

• Through such dialogue, facilitate discussions on whether new care pathways and facilities are needed prior to launch, as well as how new diagnostic and prognostic tests will be funded, especially if there is still fragmentation in funding care.

• Where pertinent and feasible, seek partnerships between health authorities, academic institutions and commercial organizations to accelerate developments that can improve care at reduced costs - especially through greater use of generic therapies.

Peri-launch

• If necessary, to adjust the process of HTA and other assessment bodies to robustly handle the diagnostic component of new targeted treatments.

• To consider developing new quality indicators around new targeted therapies together with key stakeholder groups. This builds on existing processes. This should include their assessment in practice acknowledging that any indicators developed must have validity in terms of content, face, concurrence, construct and prediction.

• To seek to include new indicators in any new guidance and guidelines associated with new targeted treatments, as well as potentially to consider their inclusion in any ongoing financial incentive schemes for physicians.

• To be critical of any proposed risk-sharing arrangements including targeted therapies and biomarkers and to be mindful of the potential administration costs. However, also aware that such arrangements post-launch could facilitate reimbursement and funding of new premium-priced drugs.

• Continually checking likely launch dates for new treatments with the relevant pharmaceutical companies to improve financial planning, especially given the premium prices requested for new targeted treatments.

Post-launch

• To integrate regular reviews of any reimbursement, funding or guidance especially as more data becomes available.

• To monitor physician adherence to any agreed guidance or reimbursement restrictions for new targeted treatments.

• To instigate additional demand-side measures such as educational initiatives and financial incentives if needed where there are concerns with adherence rates to any agreed guidance or subpopulations.

The second stakeholder group includes HTA units [27,33,98,99,228-231]. The major issues for HTA units include:

General (in addition to providing critical input peri-launch including the sensitivity and specificity of new diagnostic and prognostic tests)

• To develop and refine new methodological approaches that take into account potential changes in clinical trials and increasing use of models in systems biology-based personalized medicine approaches - especially around defining subpopulations.

• Possibly to include progression of constructive technology assessments until more data become available. However, to be mindful of concerns with surrogate data.

• Possibly to involve HTA units with discussions to modify the legal framework as well as regulatory and approval processes as more information regarding personalized medicines become available.

Post-launch

• To assist with post-launch follow-up of drugs particularly to reassess product safety in routine clinical care, as well as to provide guidance where concerns.

The third stakeholder group includes research institutions, research groups and professional medical, pharmaceutical and educational societies. The major issues for this group include:

• To focus and promote comprehensive critical research and education to understand and explore the benefits and risks with personalized diagnostic and treatment strategies.

• To assist with policy analysis and involvement in education on issues relating to personalized medicine among specialists, researchers and in the public area.

The fourth stakeholder group are physicians [3,9,34,49,60,61,107,109],[116,184-187,205,212,217,221,232]. The major issues for physicians include:

General

• To provide independent advice into clinical trial design for new biomarkers that are disease based; alternatively aimed at differentiating patients or populations based on either differences in drug metabolism, drug transporter capacity or receptor variants.

• To help design trials that improve our understanding of the sensitivity and specificity of new diagnostic tests, thereby reducing the uncertainty with their use. Such studies could include cohort studies with samples and data collected prospectively. Nested case–control studies are also potentially useful so long as blinding is maintained.

• To assist with the design of technology platforms and mathematical models that help with future decision making for individual patients as the complexity of biological systems unfold. By doing so, to improve the translation of research results into clinical practice.

• To push for ongoing independent reinterpretation of the implications of genetic tests and therapies in the light of new discoveries. This will be achieved through using trained clinical pharmacologists and physicians specializing in areas such as molecular oncology. This builds on the current controversies surrounding the pre-testing of patients prescribed clopidogrel or warfarin.

• To help translate the language of genomics into lay language to assist patients with their decision making, including the benefit:risk ratio of treatments. This will necessarily include improved knowledge of genomics among physicians from current low rates.

Pre-launch

• To work with health authorities and health insurance companies pre-launch to critically review new targeted treatments, especially where there are concerns about their potential value in practice.

• As part of this, to provide guidance to health authorities and health insurance companies about potential new quality indicators.

• To provide input into discussions on the potential value of new pharmacogenetic tests that optimize the use of new drugs post-launch especially where there are considerable uncertainties regarding their clinical value.

Peri- and post-launch

• To assist with the design of any patient registries or expansion in Electronic Health Records prior to launch, and follow this up after launch building on the experiences with, for instance, natalizumab.

• To help authorities critically assess proposed risk sharing arrangements, especially regarding the potential administrative burden.

• To assist hospital and ambulatory care Drugs and Therapeutic Committees with critically evaluating new targeted treatments, as well as to promote interface arrangements to improve the co-ordination of care between primary and secondary care physicians.

• To help with the development of educational materials for physicians and patients peri- and post-launch based on agreed guidance.

The fifth stakeholder group are patients and patient groups [27,107,109,211,212]. The major issues for this group include:

General

• To support the development of patient registries and electronic record systems that help identify patients with specific genotypes to improve their care in the future.

• Where pertinent, to work with all key stakeholder groups regarding potential goals for the sensitivity and specificity of new molecular and diagnostic genetic tests alone or in combination to reduce uncertainty with their use, especially if there is reluctance to fund ‘coverage with evidence’ schemes.

• To help authorities and physicians involved in the development of personalized medicine translate the results of research findings into lay language to assist patients with future decision making.

• To seek to be an integral part of national discussions concerning the ethics and implications of genetic testing for other family members.

• To help authorities incorporate personalized medicine into patient education schemes to enhance their understanding of this complex field for better informed discussions with physicians.

• To work with authorities to make sure that patients’ dignities and integrities are preserved with greater knowledge of their genetic make-up, and that specific groups are not excluded from societies (building on earlier comments).

Pre-launch

• To provide input to health authority and health insurance companies pre-launch discussions regarding key issues for new diagnostic tests or new targeted treatments from a patient’s perspective.

• To support the development of patient registries or other data collection activities around new targeted approaches; the results of which can also be used to inform future clinical trials and future decision making.

Pre- and peri-launch

• To help with the design and distribution of any patient information regarding new drugs, especially where there are potential safety issues.

• To help with the development of new quality indicators for new targeted drugs from a patient’s perspective to improve their validity.

• To provide input into the assessment of the potential value of new technologies especially where the findings, including potential biomarkers, are inconclusive.

Post-launch

• To help refine information for patients as more knowledge becomes available about new diagnostic approaches or new drugs, especially with respect to major adverse reactions and their implications.

• To help disseminate factual information to patients, especially where there are exaggerated claims unduly raising expectations among patients or where key issues regarding the potential side-effects of treatments have not been fully explained or adequately disseminated.

The last stakeholder group includes pharmaceutical and diagnostic companies

[11,13,75,88,90,91,98,99],[114,116,205,210,211,213,214,233],[234]. The major issues for this group include:

General

• To make explicit in the trial design for new genetic tests and biomarkers whether they are dealing with diagnostic or prognostic; alternatively, disease-based or patient- or population-based technologies. This acknowledges that different trial populations will be needed, as well as different performance characteristics for different tests. For example, new screening biomarkers need high specificity to avoid generating an excessive number of false positives whereas high sensitivity is needed for new prognostic biomarkers to avoid denying treatment to patients who could potentially benefit.

• To make the objectives of any trial design specific to answer key questions. This includes potential subpopulations where the health gain of new targeted drugs is greatest. It also includes designing studies to specifically answer questions about the sensitivity and specificity of new diagnostic and prognostic tests including the extent of any false positives and false negatives.

Pre-launch

• To instigate realism into corporate discussions regarding potential requested prices for new diagnostic tests or targeted treatments, acknowledging that the cost of providing tests includes both the acquisition costs as well as facility costs as resource pressures grow. This becomes even more important if multiple genetic tests are needed to plan future care.

• As part of this, to avoid the temptation to seek ‘orphan status’ for new targeted therapies as resource pressures grow. This may avoid rejection or delayed funding even with risk-sharing or patient access schemes to lower acquisition costs. This includes recognition that without targeting new products are increasingly unlikely to achieve premium prices as more standard drugs become available as generics and niche areas diminish.

• To acknowledge that the definition of orphan drug status may need redefining to smaller patient populations, especially with the increasing costs of orphan drugs and growing resource pressures.

• To seek scientific advice from relevant registration, HTA and funding bodies pre-launch on the potential need and relevance for developing markers and tests concurrently with developing new drugs, especially for small and medium-sized companies as part of their development process.

• Similarly, seek scientific advice for new drugs that require associated genetic testing to maximize their value especially for clinical trials that could include small subgroups of patients.

• To explore possible partnerships between diagnostic and pharmaceutical companies to provide a combined package at launch, for example, combined worldwide sales of trastuzumab and imatinib, both using established tests, were US$9.6 billion in 2010.

• To initiate possible discussions concerning rebates or discounts peri-launch to enhance the value of new targeted treatments - recognizing the complexities of current funding arrangements.

There are a number of issues arising from these considerations that need to be explored further. These include:

•greater cohesion of what is meant by personalized medicine and associated training

•the Human Microbiome Project

•DTC advertising

•evidence-based classification of genomic tests

•funding of new targeted tests and therapies.

#### Future direction and training

To enhance acceptance of personalized medicine, there needs to be greater cohesion of what this constitutes. The advent of the European Science Foundation recruiting a group to analyze the complex field of personalized medicine may help, especially as their objective is to provide future policy advice [1,36]. This is already leading to groups such as the European Alliance for Personalised Medicine issuing five key major action points for policy makers, politicians and regulators across Europe to accelerate the development, delivery and uptake of personalized medicine and diagnostics [235]. These include:

•ensuring a regulatory and funding environment that allows early patient access to novel and effective personalized medicine

•increasing research and development funding to develop new personalized medicines

•improving the education and training of healthcare professionals regarding personalized medicine and the various approaches

•acknowledging that new approaches may be needed for reimbursement and HTA assessment, which are required for patient access to personalized medicine and recognition of their value

•increasing awareness and understanding of personalized medicine among all stakeholder groups.

The funding of any new diagnostic facilities, as well as the instigation of patient education, may also be a challenge as the range of therapeutic options increase and become more complicated to navigate. Moreover, additional training of healthcare professionals, including their full understanding of the concepts of personalized medicine and targeted approaches, will also need to be addressed [212]. For instance, only 10% of physicians in the US in a recent survey believed they were adequately informed about pharmacogenomic testing [212]. However, we believe that as targeted therapies become more commonplace, fluency in genomics will increase and with it methodologies and training to handle the increasingly complex biological information. Mathematical models and decision support tools, together with developments in technology platforms, will help as well [5,36,123,236,237].

#### Human Microbiome Project

Following the completion of the Human Genome Project, there has been substantial growth in recent years in the Human Microbiome Project [238,239]. These developments open up new possibilities and horizons for studying how microbiome compositional and functional variations affect the effectiveness of drugs and their toxicity (pharmamicrobiomics), most notably in the gut. This includes research into how the microbiome interacts with human metabolic enzymes in the liver and intestine. Ultimately, we must understand better the future implications of Human Microbiome Project on drug therapeutics and personalized medicine [238]. Further discussions are outside the scope of this review paper.

This will be the subject of future research articles.

#### Direct-to-consumer advertising of genetic testing

One key issue that authorities will need to consider is the growth in private enterprises offering DTC genetic testing [47,119]. This builds on the knowledge that personalized sequencing can deliver some clinically useful information [47,118,119,240]. While we believe the uptake of as yet lightly regulated DTC activities by commercial companies may currently only occur in a limited number of situations, despite such services being accessible via mobile devices [119], there are concerns with their current lack of predictive value, clinical validity and utility, discordance of results between companies, and difficulties with interpretation, as well as test-related anxiety [2,16,47,118,241-245]. Other concerns include the variable quality of pre-test and post-test information and genetic counseling services, the lack of medical supervision among DTC companies, and inappropriate testing of minors by some [47,118,246,247].

Unregulated, this could be a major source of anxiety given concerns with the limited clinical utility of some of the tests to date [47,118,119,242]. This has encouraged professional bodies such as the European Society of Human Genetics and the Nuffield Council of Bioethics to jointly inform and warn healthcare professionals, health authorities and the public concerning potential problematic aspects of DTC genetic testing [118]. However, it is recognized that it is difficult to have an international legal framework to control such activities [118]. The European Directive 95/46/EC on the protection of individuals, and directive 2000/31/EC on certain legal aspects of information society services in particular relating to *e*commerce, should help address some of these concerns [118]. Whether this actually works remains to be seen, even if the authorities in France are already active in regulating individuals against DTC activities [118].

#### Evidence-based classification of genomic tests in clinical practice

As mentioned, algorithms have been developed in the US as part of the EGAPP initiative [107,110]. These were motivated largely by frustration at the lack of evidence regarding the translation of genomic discoveries into clinical practice [107,109,110,248].

Key considerations in the recommendations ranging from ‘do not use in practice’ to ‘implement in practice’ include [107,110]:

•level of evidence

•level of certainty - from low to high

•risk benefit profile - from unknown, unfavorable to favorable

•extent of additional research needed

•potential health impact

•evidence recommendations and actions.

As mentioned above, only one of the initial four genomic tests was recommended for implementation in practice using this approach [107].

#### Funding of new targeted therapies

Additional funding of specific tests for diagnosis, prognosis and directing treatment options can be a major concern among health authorities and health insurance companies (payers) as resource pressures grow, negating the potential benefits from personalizing treatment approaches [27]. This is illustrated by complex tests for patients with breast cancer costing approximately US$3,900 per test in the US, although these have been shown to reduce overall treatment costs [35,92,249]. Overall, the costs for complex tests range from US$1,000 to over US$4,000 per patient (2008 US prices) [35].

The UK is seeking to address some of these issues through establishing the UK Genetic Testing Network for single-gene disorders [35,250]. However, funding arrangements are still unclear if multiple pharmacogenetic and microarray-based tests are needed before initiating treatment [35], although this is changing [251]. This however may be less of an issue in the future if, as envisaged, the costs of these tests appreciably fall in price with their increasing use [44,201,252]. New funding mechanisms have also been developed in the UK to optimize the use of targeted drugs. For instance, the UK National Health Service agreed to pay AstraZeneca directly GB£157.20 to GB£210.00 per Epidermal Growth Factor Receptor (EGFR) test prior to treatment with gefitinib as part of the overall strategy for funding the drug [35]. This is implemented via a network of regional laboratories.

Funding of pharmacogenomic tests has been helped by investigators in Japan estimating that *KRAS* testing in selected patients with colorectal cancer before initiating cetuximab saved an estimated US$50 million per year compared to no testing [253,254]. This led to increased use of cetuximab [23]. *KRAS* testing has also be estimated to save the US health system over US$600 million per year in the cost of cetuximab [36,255]. The cost of treatment for patients with colorectal cancer has also been reduced in France with the instigation of regional centers undertaking *KRAS* resting [47]. Other authors have also shown that *KRAS* mutation testing prior to treatment with cetuximab saved costs [98,256].

However, there are concerns among payers that targeting of new drugs to small populations will lead to them being considered as ‘orphan drugs’ , potentially resulting in premium prices above those of new drugs without orphan status [113-115]. Such cases have already happened, for example, crizotinib and vemurafenib, which have been launched at approximately US$10,000 per patient per month excluding the cost of diagnostic tests and administration costs [91,257-259]. This is appreciably higher than for trastuzumab, which, when first launched, caused considerable funding concerns in some European countries [260]. This is at a time when the number of new cancer cases is expected to increase by over 60% in the next 20 years [49,92,253,260-262]. Other examples of high acquisition costs include new targeted drugs for patients with cystic fibrosis, which have been launched at over US$25,000 per month based on the concept of a targeted therapy in a selected subgroup of patients with cystic fibrosis [88]. More recently in the US, abiraterone was the only cancer drug approved by the FDA in 2011 without an orphan designation [112]. This situation can potentially lead to high acquisition drug costs, appreciably increasing the overall cost burden [113,233,251]. These concerns have been fuelled by Sanofi-Aventis in 2011 acquiring Genzyme for approximately US$20.1 billion [263,264], which built its base on Ceredase (imiglucerase) for Gaucher’s disease and subsequently new treatments for small patient populations with genetic deficiencies. These costs will need to be recouped. This has resulted in suggestions to reconsider the definition of orphan drugs discussed earlier.

## Conclusions

There should be considerable benefits to all key stakeholders with new technologies that can improve the diagnosis, prognosis and treatment of patients, reducing the number of patients needed to treat and increasing the number of patients needed to harm. In addition, this will also reduce the cost and consequences of ADRs [16,22,26,38,39,74,75] and, as a result, improve the health of patients within finite resources.

However, the complexity of biological systems means that gene mutations may not always express themselves as important phenotypic changes in disease patterns, making identification of potential biomarkers and new targeted treatments more difficult. This may explain why the promise of personalized medicine has not always translated into improvements in patient care in practice, and why only a limited number of targeted treatments are currently available and funded. This may also explain why advice about certain specific tests has been retracted as more data becomes available. This includes *CYP2D6* genotyping testing prior to initiation of tamoxifen [5,25,28-31] and *CYP2C19* genotyping testing prior to the initiation of treatment with clopidogrel [180-182].

It is recognized that for new technologies to be funded, there needs to be improved co-ordination among groups responsible for the funding of care and those evaluating new technologies. This will facilitate funding of new technologies that improve diagnosis, prognosis or subsequent care, especially if funding for new personalized technologies cross sectors [26]. This is beginning to happen as seen in France and the UK [35,191]. However, this has been the exception, typically with fragmentation and heterogeneity of funding across sectors [1,13,35], exacerbated in some cases by the need for multiple pharmacogenetic and microarray-based tests.

The envisaged reduction in the costs of pharmacogenetic tests [44,201,252] should increase the number of pharmacogenetic tests that are currently funded [265]. Earlier planning for the introduction of valued tests and targeted treatments should also facilitate their funding. New models, including potential coverage with evidence schemes [228], should also be explored further to facilitate funding. However, this will depend on resource requirements and the level of evidence initially provided [107,110,211].

A growing concern among payers is the requested price for new targeted treatments, especially if these increasingly resemble requested prices for orphan drugs, which is already happening [88,257-259]. In 2009, 22 targeted cancer therapies were approved by the FDA, with sales of US$16 billion/year in the US in 2009 alone for just five of these [11,45,103]. More recently, as mentioned, abiraterone was the only cancer drug approved by the FDA in 2011 that did not seek an orphan designation [112]. Alongside this, payers are aware of the considerable number of biological drugs in development. For example, 42% of over 600 drugs in Phase I to III in companies listed in the NASDAQ Biotech Index are biological drugs, the majority of which are for cancer or immunological diseases [266]. Overall, it is estimated that up to half of current clinical pipelines among leading companies include targeted or stratified medicines [45]. These issues and concerns need to be addressed to be able to fully fund new premium-priced targeted treatments considered valuable by payers of healthcare.

Payers across Europe are already seeking ways to release resources to help fund new technologies. This is through increased use of low-cost generics versus patented drugs in a class or related class, which will grow as more standard therapies lose their patents [83,267-273]. Commercial organizations can play their part through realistic pricing for their new technologies. This should be a possibility since developments in pharmacogenomics should reduce the number of failures, accelerate drug development and potentially reduce the number of patients needed in clinical trials through enriched patient populations [26], resulting in appreciably reduced development costs [50]. Marketing activities should be lower for targeted treatments with robust evidence, This should translate into lower prices, with US$53 billion per year spent in recent years by pharmaceutical companies in the US alone on promoting their products to physicians [274].

Governments and health authorities also need to tackle the ethical issues associated with an increasing personalized approach. These include who will own the genomic data if population-based genomic sequencing increases, as well as issues of equity if high prices persist for diagnostic and prognostic tests and for new targeted treatments [27].

In conclusion, we hope we have stimulated the debate about personalized medicine and the ways forward for all key stakeholder groups. As a result, we hope this will help translate the promise of personalized medicine into clinical practice to benefit patients in the future.

## Abbreviations

ADRs: Adverse drug reactions; DTC: Direct-to-consumer; EGAPP: Evaluation of genomic applications in practice and prevention; EGFR: epidermal growth factor receptors; FDA: US Food and drug administration; HTA: Health technology assessment; IgE: Immunoglobulin E; PARP: Poly (adenosine diphosphate–ribose) polymerase; PML: Progressive multifocal leukoencephalopathy.

## Competing interests

No author has a financial competing interest to declare. The majority of authors are employed by health authorities, health insurance companies, Physician Associations or are advisers to them working in Universities or for other organisations. These include BG (numerous health authorities across Europe), EZ-B (HVB, Austria), I G-I (Basque Country, Spain), JJ (NHS Tayside and SMC, Scotland), REM (KI and Stockholm County Council), EA (Pharmaceutical Pricing Board, Finland), MB (NHS Scotland), IB (NHS Scotland), AB (HVB, Austria), SC (Department of Health, UK), ED (Catalonia Health Service), JF (Health Insurance Institute, Slovenia), KG (National Health Insurance Fund, Lithuania), AH (Department of Health, UK), HH (Physician Association, Hessen, Germany), KHv and CK (Norwegian Medicine Agency), SJ (Horizon Blue Cross Blue Shield, New Jersey, USA), MK (Republic Institute for Health Insurance, Serbia), OL (State Agency of Medicines, Estonia), S-AL (Stockholm County Council, Sweden), AM (NHS, UK), LM (National Centre for Pharmacoeconomics, Ireland), FN (TLV, Sweden), GS (AOK Health Insurance Fund, Germany), CS (CNAM, France), US (Health Insurance Funds, Germany), DT (Health Insurance, Slovakia), VV-P (Croatian Institute for Health Insurance), LV (Ministry of Health, Croatia), MvW (Health Care Insurances, Netherlands), CZ (Barcelona Health Region, Spain).

However, the content of the paper and the conclusions are those of each author and may not necessarily reflect those of the organization that employs them.

## Authors’ contributions

BG, AFF, PKC, EZ-B, REM, LLG and RA - developed the first draft and co-ordinated activities and inputs regarding additional drafts. This included potential definitions (AEF, PKC and EZ-B) as well as authority perspectives from Sweden (REM, LLG). IG-I - provided examples, critiqued subsequent drafts, co-ordinated the development around key medical, ethical, legal, social, economic and organizational issues, and contributed to the iterative process for the development of stakeholder recommendations generally as well as for Spain. He also critiqued subsequent drafts following reviewer comments. JJ - provided input especially from a timing perspective regarding input from key stakeholder groups, critiqued subsequent drafts including those following reviewer comments. EA - inputted into the development of the paper particularly from the perspective of authorities in Finland. This included the iterative process for key issues and the development of recommendations. CB - provided input into the first drafts based on his experience with personalized medicine, especially from a regulatory perspective. In addition, critiqued subsequent drafts including the iterative process for key issues and the development of recommendations. MB, IB and KP – inputted into the development of the paper particularly from the perspective of the authorities in Scotland. This included the iterative process for key issues and the development of recommendations. AB - inputted into the development of the paper particularly from the perspective of authorities in Austria (including EZ-B) as well as generally across Europe. This included the iterative process for key issues and the development of recommendations. AF, KHa, AH and AM - inputted into the development of the paper including the perspective of authorities in the UK (nationally and regionally), as well as critiqued subsequent drafts. This included the iterative process for key issues and the development of recommendations. SC also critiqued subsequent drafts following reviewer comments as well as from the perspective of authorities in the UK (nationally and regionally). ED and CZ - inputted into the development of the paper particularly from the perspective of authorities in Spain (regional and national) as well as generally from a clinical pharmacology perspective. This included the iterative process for key issues and the development of recommendations. JF - inputted into the development of the paper particularly from the perspective of authorities in Slovenia as well as critiquing subsequent drafts. This included the iterative process for key issues and the development of recommendations. KG - inputted into the development of the paper particularly from the perspective of authorities in Lithuania, as well as critiquing subsequent drafts. This included the iterative process for key issues and the development of recommendations. MG - inputted into the development of the paper particularly from the perspective of authorities in Portugal. This included the iterative process for key issues and the development of recommendations. HH, GS, US - inputted into the development of the paper particularly from the perspective of Sickness Funds, Physician Associations and their advisers in Germany. This included the iterative process for key issues and the development of recommendations. KHv, CK - inputted into the development of the paper particularly from the perspective of the national authorities in Norway and critiqued subsequent drafts. This included the iterative process for key issues and the development of recommendations. SJ – inputted into the paper following reviewer comments - particularly from the perspective of managed care organizations in the US. This included the iterative process for key issues and the development of recommendations. MK - inputted into the development of the paper particularly from the perspective of the authorities in Serbia and critiqued subsequent drafts. This included the iterative process for key issues and the development of recommendations. OL - inputted into the development of the paper particularly from the perspective of the authorities in Estonia. This included the iterative process for key issues and the development of recommendations. S-AL, FN - inputted into the development of the paper particularly from the perspective of authorities in Sweden (national and regional) - building on comments from REM and LLG. This included the iterative process for key issues and the development of recommendations. KM, MW - inputted into the development of the paper particularly from the perspective of the various authorities in Poland including critiquing subsequent drafts. This included the iterative process for key issues and the development of recommendations. LM - inputted into the development of the paper particularly from the perspective of authorities in Ireland as well as generally using her health economic background. This included the iterative process for key issues and the development of recommendations. CS - inputted into the development of the paper particularly from the perspective of the various national authorities in France. This included the iterative process for key issues and the development of recommendations. SS - inputted into paper following reviewer comments based on his experience as well as generally from the perspective of authorities in Belgium. This included the iterative process for key issues and the development of recommendations. DT - inputted into the development of the paper particularly from the perspective of the national authorities in Slovakia. This included the iterative process for key issues and the development of recommendations. VV-P and LV - inputted into the development of the paper particularly from the perspective of the authorities in Croatia as well as critiqued subsequent drafts. This included the iterative process for key issues and the development of recommendations. MvW - inputted into the development of the paper based on his experience with quality indicators as well as generally from the perspective of authorities in the Netherlands. This included the iterative process for key issues and the development of recommendations. DW-R - inputted into the development of the paper particularly from the patients’ perspective based on her considerable experience in this area. This included he iterative process for key issues and the development of recommendations. All authors read and approved the manuscript.
